# Design, Experiments and Simulation of Voltage Transformers on the Basis of a Differential Input D-dot Sensor

**DOI:** 10.3390/s140712771

**Published:** 2014-07-17

**Authors:** Jingang Wang, Can Gao, Jie Yang

**Affiliations:** State Key Laboratory of Power Transmission Equipment & System Security and New Technology, Chongqing University, Chongqing 400044, China; E-Mails: jingang@cqu.edu.cn (J.W.); 20131102043@cqu.edu.cn (J.Y.)

**Keywords:** electric field simulation, D-dot voltage probe, differential input, non-contact measurement, Ansoft Maxwell

## Abstract

Currently available traditional electromagnetic voltage sensors fail to meet the measurement requirements of the smart grid, because of low accuracy in the static and dynamic ranges and the occurrence of ferromagnetic resonance attributed to overvoltage and output short circuit. This work develops a new non-contact high-bandwidth voltage measurement system for power equipment. This system aims at the miniaturization and non-contact measurement of the smart grid. After traditional D-dot voltage probe analysis, an improved method is proposed. For the sensor to work in a self-integrating pattern, the differential input pattern is adopted for circuit design, and grounding is removed. To prove the structure design, circuit component parameters, and insulation characteristics, Ansoft Maxwell software is used for the simulation. Moreover, the new probe was tested on a 10 kV high-voltage test platform for steady-state error and transient behavior. Experimental results ascertain that the root mean square values of measured voltage are precise and that the phase error is small. The D-dot voltage sensor not only meets the requirement of high accuracy but also exhibits satisfactory transient response. This sensor can meet the intelligence, miniaturization, and convenience requirements of the smart grid.

## 1. Introduction

The accuracy, convenience, and rapid response of a voltage transformer serve an important function in power system fault analysis, power system monitoring, and electric energy measurement of smart grid. Potential transformers (PTs) and capacitive voltage transformers (CVTs) are commonly used in high-voltage and extra-high-voltage grids in China. However, PTs are confronted with problems like large volume and increasing insulation difficulty as voltage grade increases [1]. Compared with PTs, CVTs have more advantages due to their simple insulation structure and high performance-price ratio. However, CVTs have poor transient responses because they contain coupling capacitances, intermediate transformers, compensation reactors, and other energy storage elements. Moreover, high-frequency oscillation caused through ferroresonance easily occurs on the secondary side, which will threaten the safe operation of the apparatus [2]. Optical voltage transformers (OVT) based on the Pockels Effect have numerous advantages, such as high measuring accuracy, good safety, and avoidance of electromagnetic field interference, however, changes of external temperature significantly affect the reliability and measurement accuracy of OVTs. Moreover, OVTs based on the Pockels Effect have the disadvantage of a nonlinear problem in photoelectric conversion [3].

### 1.1. Traditional D-Dot Voltage Sensor

**Figure 1.**
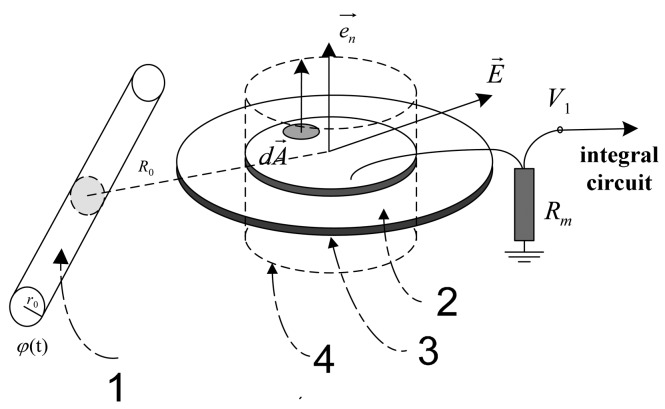
Theory of the traditional D-dot probe.

The D-dot output voltage signal is proportional to the first-order micro component of the electric displacement vector in the space of sensor to time Ḋ or εdE/dt;, which is thus called a D-dot voltage sensor. Traditional D-dot voltage sensors based on the electric field coupling principle can be used to measure high voltage levels of up to 220 kV in theory. A D-dot sensor has a simple structure, as shown in Figure 1. Thus, the frequency response of such sensor is equal to a first-order RC circuit [4].The circuit component parameters are needed for adjustment until the bandwidth satisfies the measurement range from several Hertz to 6 × 10^5^ Hz, which is significantly larger than that of a traditional voltage sensor [5].

#### Principle of the Traditional D-Dot Sensor

Figure 1 shows the structural diagram of a traditional D-dot voltage sensor. Part 1 of Figure 1 shows the measured wire. Part 2 of Figure 1 shows the metal conductor with high electrical conductivity. Part 3 of Figure 1 shows the grounding insulation. Part 4 of Figure 1 shows the closed gauss surface, which is not a physical part of the sensor. The traditional D-dot voltage sensor incorporates the grounded insulator and measuring resistance *R_m_*. The insulator connects to a coaxial cable and a metal conductor with high electrical conductivity. The electrode near the measured conductor 1 can induce the charge on the basis of the electric field coupling principle [6]. A closed Gaussian surface exists on the metal conductor surface. Gauss' theorem is applied to the closed Gaussian surface:

(1) $$q=\oint_{A}{ɛ}_{0}\vec{E}\cdot d\vec{A}={ɛ}_{0}\vec{E}A_{ɛq}$$

where *E⃗* is the electric field strength of the measured point, *A_εq_* is the equivalent area of the sensor, and *ε*_0_ is permittivity of air.

When the changed induced charge flows through the measuring resistor, such a charge will generate the resistive voltage drop *V*_1_(*t*) on the measuring resistor. As a result, the measured wire potential *φ*(*t*) becomes proportional to the electric field intensity of the sensor measuring point [7–9]. Thus, the relationship between the single electrode and measured voltage is:

(2) $$V_{1}(t)={ɛ}_{0}A_{ɛq}R_{m}\frac{d}{dt}E(t)=\frac{{ɛ}_{0}A_{ɛq}R_{m}r_{0}}{{R_{0}}^{2}}\frac{d}{dt}\varphi (t)$$

Thus, the wire voltage is given by:

(3) $$\varphi (t)=\frac{{R_{0}}^{2}}{{ɛ}_{0}A_{ɛq}R_{m}r_{0}}\int V_{1}(t)dt$$

where *R*_0_ is the distance between the measured wire and sensor, and *r*_0_ is the wire radius.

**Figure 2.**
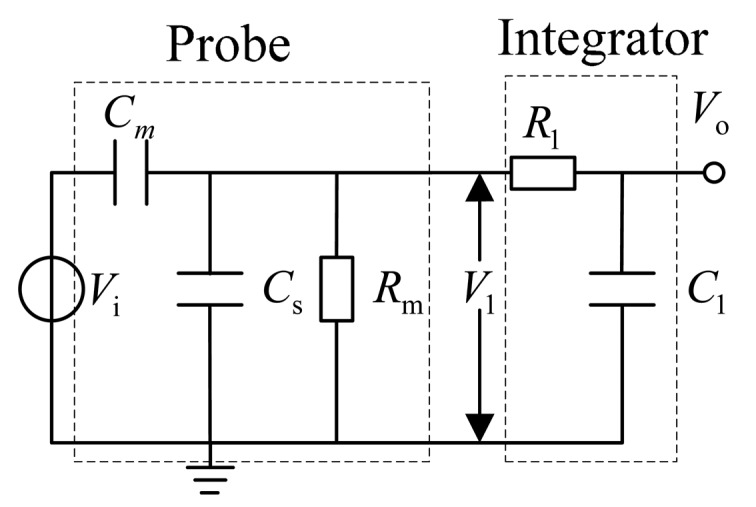
Equivalent circuit of the traditional probe and passive integrator.

The D-dot output signal *V*_1_(*t*) should be integrated into Equation (3) to obtain the time domain waveform. Figure 2 shows the equivalent measurement circuit of the traditional D-dot voltage sensor [10].

In Figure 2, *V_i_* is the measured voltage, *C_m_* is the equivalent mutual capacitance between the sensor and measured wire, and *C_s_* is the equivalent stray capacitance between the sensor and the earth [11]:

(4) $$H(s)=\frac{V_{i}}{V_{1}}=\frac{C_{m}R_{m}s}{(C_{m}+C_{s})R_{m}s+1}$$

Equation (3) shows that the value of (*C_m_* + *C_s_*) is *pF* level, where *R_m_* cannot reach 10^12^ Ω when (*C_m_* + *C_s_*)*R_m_* ≪ 1, such that the transfer function in Equation (4) is given by:

(5) $$H(s)=\frac{V_{i}}{V_{1}}\approx C_{m}R_{m}s$$

**Figure 3.**
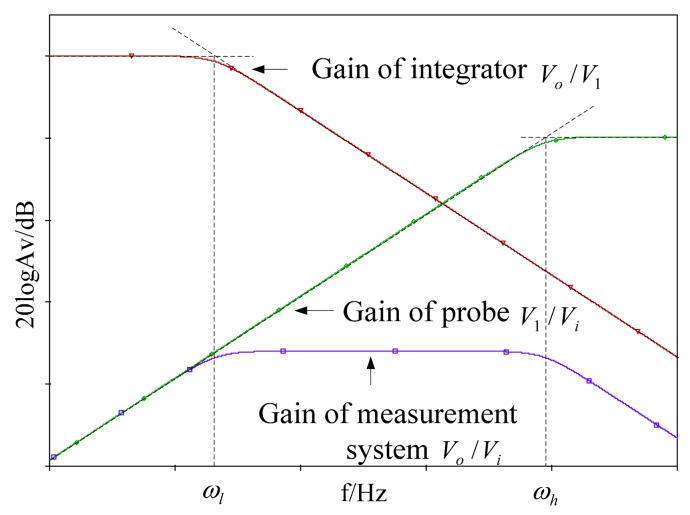
Bode plot of the traditional D-dot probe.

Thus, traditional D-dot sensor will work in a differential pattern. Figure 3 shows the Bode plot of the traditional D-dot probe [12].

In Figure 3, the upper limit of the D-dot measurement bandwidth is restricted by the angular frequency of the D-dot measuring circuit, whereas the lower limit of the D-dot measurement bandwidth is restricted by the angular frequency of the D-dot integration circuit [13,14]. The use of an integrator hinders the improvement of the signal-to-noise ratio of the sensor. Meanwhile, the waveform is distorted by the stray parameter of the integrator [15].

### 1.2. Improved Differential Input of D-Dot Sensors

**Figure 4.**
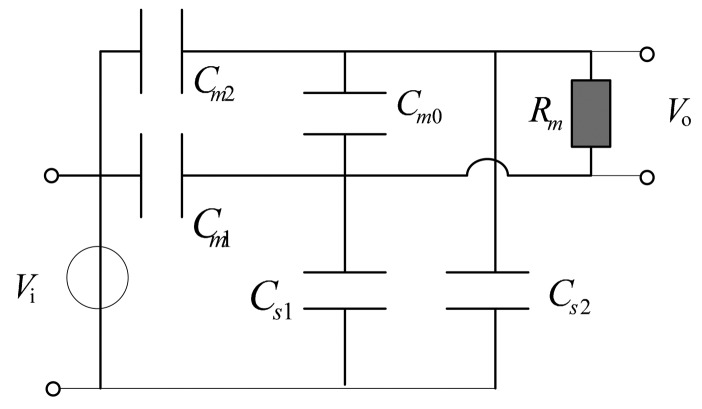
Equivalent circuit of the probe and passive integrator.

For the sensor to work with a self-integrating pattern, the differential input pattern is adopted for circuit design. The differential voltage signal is obtained by measuring the floating potential difference of both ends of the electrode. Grounding is then removed. Figure 4 shows the new-designed differential D-dot sensor that can measure the grid power line without contact and the need of ground insulation where *C_m_*_2_ and *C_m_*_1_ are the mutual capacitances between the annular electrode and measured transmission conductor, *C_s_*_1_ and *C_s_*_2_ are stray capacitances between two annular electrodes and the earth *C_m_*_0_ is the mutual capacitance of two annular electrodes, and *R_m_* is the input equivalent resistance of the differential amplifier for measurement.

The transfer function, amplitude-frequency characteristic of the improved D-dot sensor are given by the following expressions.

The transfer function is:

(6) $$H(s)=\frac{V_{o}}{V_{i}}=\frac{sR_{m}C_{1}}{sR_{m}C_{2}+1}$$

The amplitude-frequency characteristic is:

(7) $$\left|H(\omega )\right|=\frac{R_{m}+C_{1}}{\sqrt{{\left(R_{m}C_{2}\right)}^{2}+\frac{1}{\omega^{2}}}}$$

The phase-frequency characteristic is:

(8) $$\begin{array}{c} ∠H\left(\omega \right)=\arctan\frac{1}{R_{m}C_{2}\omega }\\ C_{1}=\frac{C_{m1}C_{s2}-C_{m2}C_{s1}}{C_{m1}+C_{m2}+C_{s1}+C_{s2}}\\ C_{2}=\frac{1}{\frac{1}{C_{m1}+C_{s1}}+\frac{1}{C_{m2}+C_{s2}}}+C_{m0} \end{array}$$

where *ω* is angular frequency of the D-dot sensor. The value of *R_m_* can reach the 10 GΩ level. Equations (7) and (8) show that, when *C_m_*_0_ is increased to reach the μF level through paralleling capacitances, the value of *R_m_C*_2_ will be significantly greater than 1/*ω*. Meanwhile, the input and output of the sensor are unrelated to frequency. In addition, the sensor can work in the self-integrating pattern over the whole frequency band without the need for an additional integrator.

In conclusion, the traditional D-dot voltage sensor has been improved. The differential input D-dot sensor can not only add the frequency bandwidth, but also avoid actual electrical connection with the ground electrode from input to output, thus preventing the damage to the secondary side when grounding current flows through the measurement system. The problem of ground insulation will not necessarily be aconcern. The new and improved D-dot sensor also has the advantages of miniaturization and non-contact digital measurement.

### 1.3. Model Design and Simulation of Improved D-dot Sensor

#### Model Design of the D-Dot Sensor

**Figure 5.**
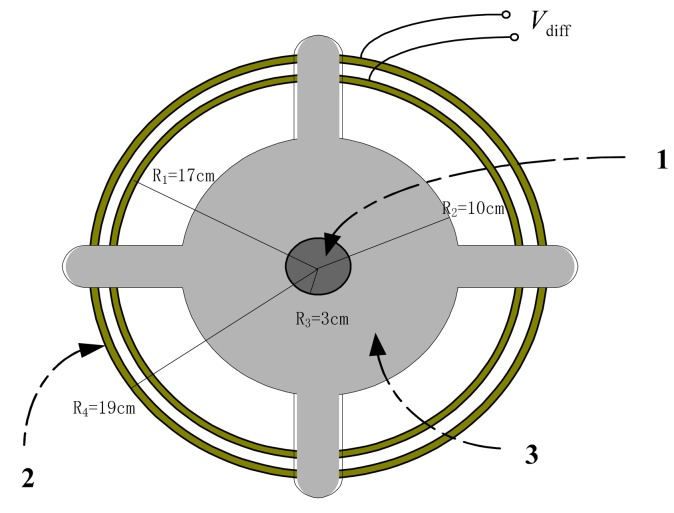
Structure of the improved D-dot probe.

Figure 5 shows the hardware architecture of the improved D-dot sensor. This architecture mainly consists of an inner copper ring, an exterior copper ring, and an insulating support. The inner and exterior copper rings are concentric annuli of different radii. The rings are both fixed on the insulation support. A through hole is used to fix the measured wire onto the insulating support, which is made of epoxy resin with added E-type fiberglass. The two copper rings can be considered as two D-dot sensors with different *A_εq_*. Epoxy resin not only supports the whole sensor structure, but also reduces the influence of the external electric field owing to its good insulation characteristic (the electric breakdown strength of E-type fiberglass epoxy resin is 35,000 kV/m). Finally, the insulation capability of the entire sensor is improved, and output power is reduced to satisfy the miniwatt drive requirements for there lay protection of the power system and of the secondary measuring equipment.

where: 1 is the measured wire, 2 denotes the copper rings, and 3 is the epoxy resin support.

## 2. Simulation of Improved D-Dot Sensor

### 2.1. Circuit Simulation of the Improved D-Dot Sensor

Through the optimization function of Ansoft Maxwell, the values of *C_m_*_0_, *C_m_*_1_, *C_m_*_2_, *C_s_*_1_, and *C_s_*_2_ can be calculated. The specific values are shown in Table 1.

**Figure 6.**
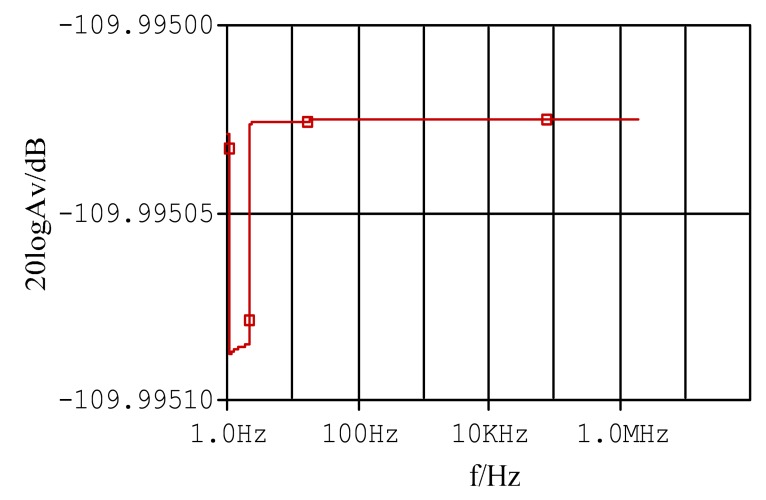
Bode plots of self-integrating D-dot probe.

PSpice simulation software is used to prove that the measurement of the improved sensor has greater bandwidth than previously achieved. Figure 6 shows that steady gain can be sustained with a large bandwidth.

### 2.2. Simulation Model

**Figure 7.**
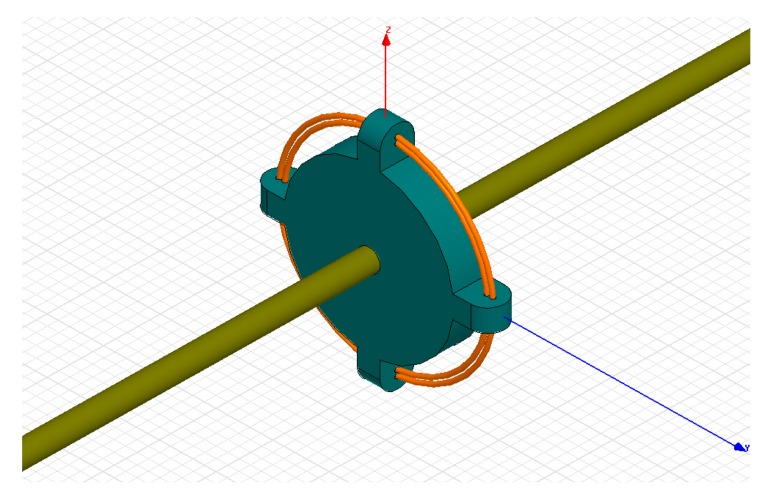
Simulation model of D-dot probe.

Figure 7 shows the 3D finite element model established by the electromagnetic simulation software Ansoft Maxwell. The modeling steps are given below.

The excitation source can be approximated to an infinitely long transmission line, and the electric field vector is set to 0 at infinity from the excitation source when considering the actual operating conditions of the sensor [16]. Thus, the calculation method for expanding the electric field area is employed to solve the open electric field problem. For the model of the D-dot sensor, the distribution of the electric field is axisymmetric. Thus, so the cylindrical coordinate system (*r*,*φ*,*z*) is adopted. In this system, *r* is the coordinate of radius, *φ* is the azimuth angle, and *z* is the symmetry axis coordinate. In the model, the half fields are chosen as the calculation field. In the whole calculation field of the model, the potentiometric function satisfies the following differential equation [17]:

(9) $$\frac{1}{r}\frac{\partial }{\partial r}(r\frac{\partial \varphi }{\partial r})+\frac{\partial^{2}\varphi }{\partial^{2}z}=0$$

The critical region between the epoxy resin structure of the D-dot sensor and external air satisfy the equations:

(10) $$\{\begin{array}{l} \varphi_{1}=\varphi_{2}\\ {ɛ}_{0}\frac{\partial \varphi_{1}}{\partial n}={ɛ}_{1}\frac{\partial \varphi_{2}}{\partial n} \end{array}$$

where *ε*_0_ is the air relative permittivity, *ε*_1_ is the dielectric constant of epoxy resin, and *n* is the outer normal of the interface [18].

### 2.3. Results and Analysis of Simulation

To demonstrate the feasibility of the structural design and material selection of the D-dot voltage sensor and to solve the problems of electric field distortion and ground insulation, the electric field distribution curves around the sensor at a 10 kV power frequency voltage are simulated.

**Figure 8.**
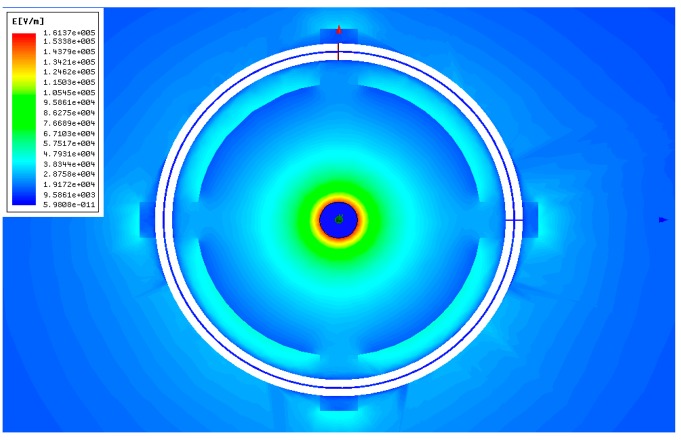
Electric field distribution of D-dot probe.

The electric field intensity inside the sensor decreases with increasing distance, as shown in Figure 8. In addition, the linearity of the relation curves between electric field intensity and distance is relatively ideal, which indicates that the influence from the sensor with the highest electric field intensity inside the epoxy resin support is 161.37 kV/m. This value is significantly smaller than the breakdown strength of epoxy resin. This finding demonstrates that the material and structural design of the support will not be broken down by a high electric field, thus improving the insulating strength of the sensor.

**Figure 9.**
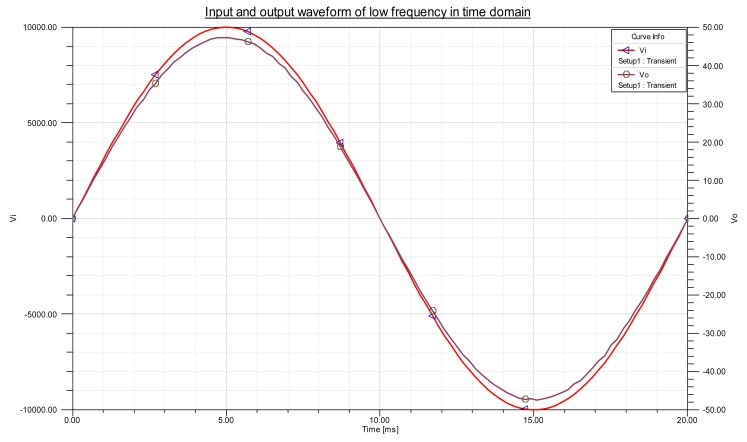
Input and output waveform of the sensor.

To demonstrate that the improved sensor has no phase delay in steady state, the time domain waveforms of the input and output sensors at 50 Hz sine excitation are simulated. In Figure 9, *V_i_* is the measured voltage, whereas *V_o_* is the output voltage of the sensor. As shown in Figure 9, almost no phase delay exists between *V_i_* and *V_o_*.

## 3. Performance Test and Data Analysis of Sensor

After designing and establishing the D-dot voltage sensor model, the steady-state error and transient response experiments are conducted to evaluate model performance.

**Figure 10.**
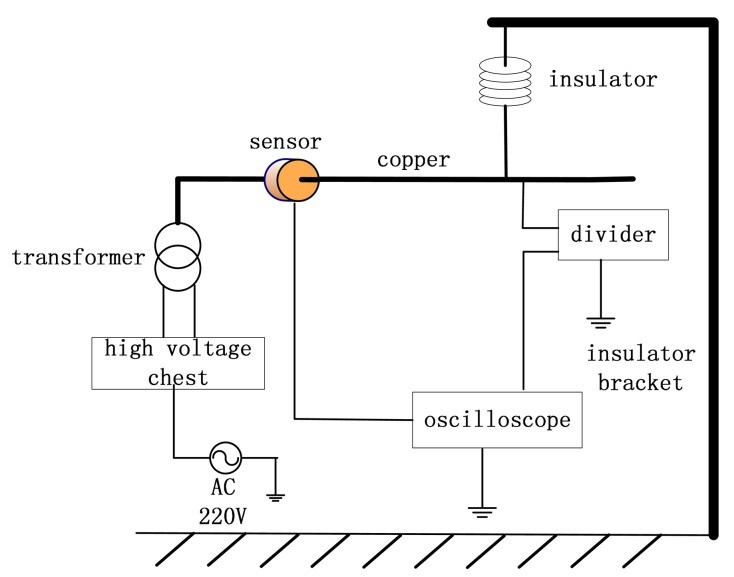
Test platform of high voltage.

The high-voltage test platform is shown in Figure 10. The voltage control box controls the power frequency voltage output. The transformer connects to a long straight copper rod that is taken as a single-phase transmission wire [19,20]. The high-voltage probe, which has a compensating circuit, is used to measure the voltage of the wire, which goes through the center of the sensor. The relative amplitude of the waveforms will be compared on the oscilloscope by measuring the waveform of the output voltage of the sensor and the wire simultaneously. The polder model is employed for the measurement oscilloscope. This model can mitigate the interference signal and improve electromagnetic compatibility. The attenuation ratio of the high-voltage probe is 1000:1, and the probe type is a Tektronix P6015A. The base and vertical accuracies are 0.75% and 1.5%, respectively. This probe can rectify and compensate the measured voltage to eliminate measurement errors. Therefore, the output of this probe can be used as standard contrast signal of the experiment.

### 3.1. Steady-State Error Experiment

A voltage of 10 kV is used as the standard measuring voltage, and the transformer is controlled for the wire voltage to reach 10%, 20%, 50%, 80%, 100%, and 120% of 10 kV to measure the output voltage of the high-voltage probe and sensor. Subsequently, the output voltage values are calculated to determine the ratio error. As shown in Table 2, *U_HV_* is the voltage of the high-voltage probe, which is converted to the primary side; and *U_D-dot_* is the measured sensor voltage.

where Ratio Error (*ε*%) is given by $ɛ\%=\frac{K_{n}U_{D-\text{dot}}-U_{HV}}{U_{HV}}\times 100\%$.

**Figure 11.**
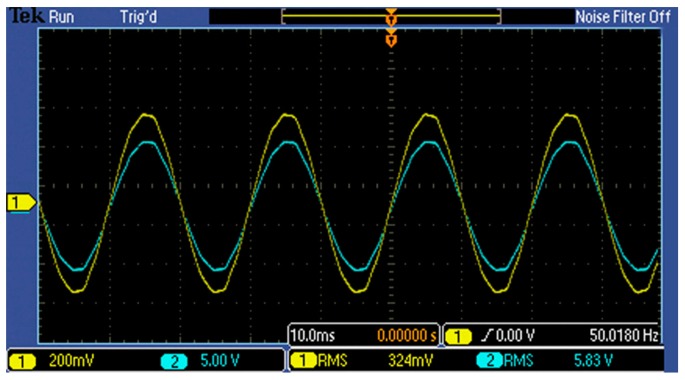
10/ $\sqrt{3}$ kV AC voltage waveform comparison.

**Figure 12.**
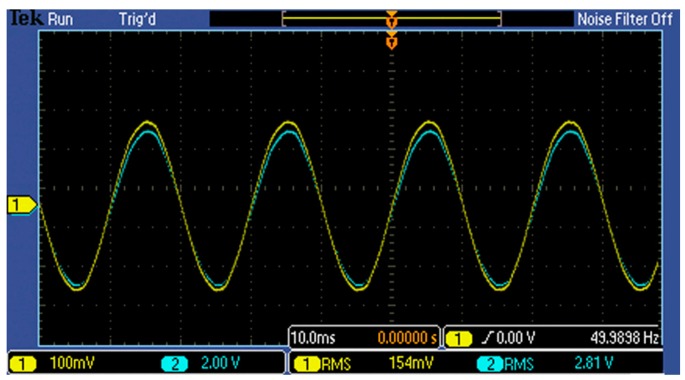
5/ $\sqrt{3}$ kV AC voltage waveform comparison.

The diagram below is based on a 10 kV power frequency voltageand depicts acomparison of the voltage waveform between a high-voltage probe (CH2, blue) and that of sensor (CH1, yellow) when the wire is applied with a phase voltage of 10 and 5 kV successively. In Figures 11 and 12:

Figures 11 and 12, as well as Table 2, illustrate that:

1. The measured voltage in Table 2 has been fitted once, and its square error is 0.014, which indicates that the sensor can maintain a linear input-output trait within the range of 10% to 120% *U_n_* and has a satisfactory dynamic range.
2. Table 2 shows that within the range of 10% *U_n_* to 120% *U_n_*, the ratio error is less than 1%, which proves that the sensor precision is relatively high and that the RMS value error is relatively small.
3. Figures 11 and 12 illustrate that the error of the waveform phase of the sensor and high-voltage probe, as well as the degree of waveform distortion, is very small.
4. As seen from Table 2, the transformation ratio of the D-dot sensor is approximately 1:2203.

### 3.2. Experiments of Transient Response

The D-dot sensor implements voltages through electric field coupling and does not contain inductive components. Therefore, the response of such sensor to the transient voltage waveform is relatively fast. An impulse voltage test is conducted to verify the transient performance of this sensor.

**Figure 13.**
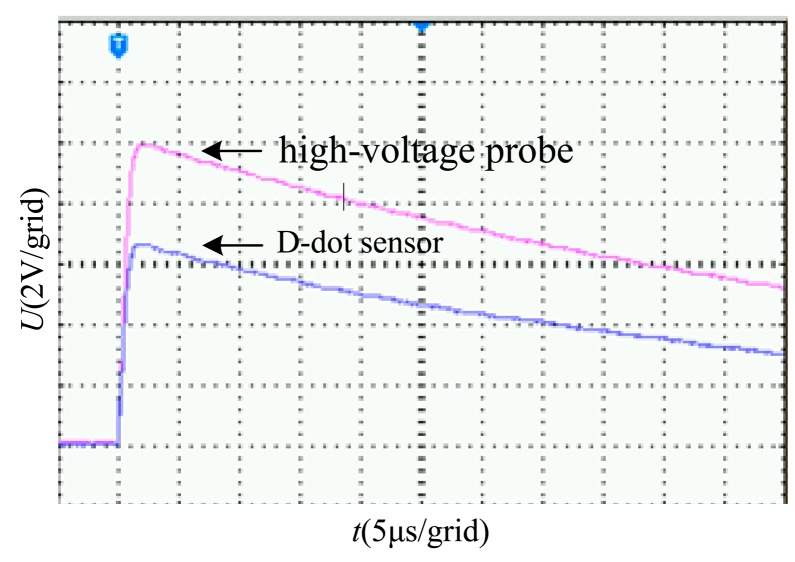
Transient oscillogram of 1.2/50 μs lightning wave.

The 1.2/50 μs standard lightning impulse voltage is applied to the line with an impulsive voltage generator, and the signal measured by the sensor and high-voltage probe were displayed on the oscilloscope, as shown in Figure 13.

As shown in Figure 13, the D-dot voltage sensor exhibits rapid response speed while preventing the occurrence of high-frequency oscillation.

## 4. Conclusions and Outlook

A new kind of differential inputs D-dot voltage sensor was designed to measure the transmission line voltage. The designed model was theoretically analyzed and simulated. In addition, steady and transient state experiments were conducted. The simulation and experimental results indicate that the sensor possesses tiny steady-state error and excellent transient performance. In this method, no electrical connection exists between the sensor and transmission line. The new sensor achieves miniaturization as well as non-contact digital measurement and is not needed to be insulated against ground, adapting to the development of smart grid and offering a new approach for electric parameters measurement of power system.

In future research, the measuring error will be analyzed and electromagnetic interference, variation of temperature and relative humidity will be taken into consideration to make sure the sensor can effectively work in field operation. Furthermore, the decomposition problem of three-phase composite electric field will be researched to achieve three-phase voltage measurement.

## Figures and Tables

**Table 1.** Capacitance parameters of a single.

*C*_*m*1_/*pF*	*C*_*m*2_/*pF*	*C*_*s*1_/*pF*	*C*_*s*2_/*pF*	*C*_*m*0_/*pF*
17.6425	15.7360	69.5856	94.0904	5631

**Table 2.** Accuracy test result of D-dot sensor.

**Measuring Point**	**U_HV_/kV**	**U_D-dot_/V**	**Ratio Error(%)**
10% U_n_	0.99	0.453	0.90
20% U_n_	2.00	0.915	0.75
50% U_n_	4.98	2.29	0.52
80% U_n_	8.02	3.68	0.31
100% U_n_	10.03	4.60	0.26
120% U_n_	12.00	5.50	0.19
